# The Vasomotor Impact of Cu/ZnSODs Is Higher in Arterial Smooth Muscle of Early Postnatal Rats Compared to Adult Animals

**DOI:** 10.3390/antiox14101231

**Published:** 2025-10-14

**Authors:** Anastasia A. Shvetsova, Valentina S. Shateeva, Dmitry S. Semenovich, Rudolf Schubert, Dina K. Gaynullina

**Affiliations:** 1 Faculty of Biology, M.V. Lomonosov Moscow State University, Moscow 119234, RussiaGaynullinaDK@my.msu.ru (D.K.G.); 2 A.N. Belozersky Institute of Physico-Chemical Biology, Moscow State University, Moscow 119992, Russia; 3 Physiology, Institute of Theoretical Medicine, Faculty of Medicine, University of Augsburg, 86135 Augsburg, Germany

**Keywords:** superoxide dismutase, reactive oxygen species, diethyldithiocarbamate, artery, smooth muscle, vascular tone, endothelium, early postnatal period

## Abstract

The mechanisms of vascular tone regulation during early postnatal ontogenesis differ considerably from those in adulthood. We tested the hypothesis that the vasomotor influence of superoxide dismutases is higher in arteries of rats shortly after birth compared to adult animals. Saphenous arteries of 10 to 15 day (“young”) and 3 to 4 month (“adult”) old rats were studied using quantitative PCR, spectrophotometry, Western blotting, and isometric myography. *Sod3* mRNA was most abundant in both adult and young saphenous arteries. Total SOD activity, Cu/ZnSODs activity, and SOD3 protein content were higher in young compared to adult arteries. H_2_O_2_ caused vessel contraction, while elimination of H_2_O_2_ weakened contractile responses of endothelium-denuded young arteries. The Cu/ZnSOD inhibitor DETC had no influence on contraction of adult arteries, but weakened contraction of endothelium-denuded, but not endothelium-intact arteries of young rats. The latter effect was observed in the presence of the NO-synthase inhibitor L-NNA, but not with the soluble guanylate cyclase inhibitor ODQ. DETC had no effect in the presence of sodium nitroprusside. Thus, Cu/ZnSODs promote contraction of saphenous arteries in the early postnatal period, but not in adult age. This influence of Cu/ZnSODs is counteracted by endothelial NO in early postnatal arteries.

## 1. Introduction

Superoxide dismutases (SODs) are enzymes that catalyze the conversion of the highly reactive superoxide anion radical O_2_^•−^ into a more stable and less aggressive molecule, hydrogen peroxide H_2_O_2_. H_2_O_2_ has a number of signaling functions in the cell, including vasomotor effects [1]. Notably, H_2_O_2_ may cause both relaxation or contraction of arterial smooth muscle, which depends on many factors, including the animal species, the artery type, the presence of endothelium, etc. [2,3,4,5,6].

There are three isoforms of SODs: cytoplasmic Cu/ZnSOD (SOD1), mitochondrial MnSOD (SOD2), and extracellular Cu/ZnSOD (SOD3) [1]. All three isoforms are expressed in blood vessels. However, among the isoforms of SOD, the functional roles of SOD1 and SOD3 in the regulation of arterial smooth muscle tone have been the most studied due to the existence of their inhibitor diethyldithiocarbamate (DETC) [7], which chelates Cu^2+^ ions in the active centers of the enzymes. Thus, in rat aorta, DETC did not affect phenylephrine-evoked contractile responses [8,9]. However, DETC caused an increase of norepinephrine-induced contractile responses of mouse aorta [10] and rat mesenteric arteries [11], as well as serotonin-induced contraction of mouse aorta [12], which may indicate an anticontractile role of Cu/ZnSOD-produced H_2_O_2_ in several arteries. It is worth noting, however, that such effects were reported for arteries from mature animals.

Nevertheless, the mechanisms of smooth muscle tone regulation, including those involving reactive oxygen species (ROS) [13], are considerably different between adulthood and the period of early postnatal ontogenesis. However, the functional contribution of SODs to the regulation of arterial tone in the early postnatal period has been poorly studied and the few existing studies focus mainly on the pulmonary circulation. For example, it was shown that DETC increased U46619-induced contractile responses of pulmonary arteries from newborn and two-week-old piglets [14]. At the same time, SOD content/activity in lung tissue of newborn or early postnatal organisms (human, rabbit, and mouse) were lower compared to adults [15,16,17] which might be associated with a smaller functional contribution of SODs in the pulmonary circulation at the early postnatal period compared to adulthood. However, the vasomotor impact of SODs on the regulation of smooth muscle contraction in systemic arteries, which play a key role in the regulation of systemic blood pressure and blood flow distribution during the early postnatal period, has not been studied yet, to the best of our knowledge. The need and significance to address this issue also follows from the widely used antioxidant therapy of newborns in the clinic [18], which does not take into account the probable consequences of the vasomotor role of SODs.

Therefore, in this study we hypothesized that the functional impact of SODs (mainly SOD1 and SOD3) is different in systemic arteries of early postnatal rats and adult animals.

## 2. Materials and Methods

### 2.1. Animals and the Main Object of the Study

Animal studies are reported in compliance with the ARRIVE guidelines 2.0 [19,20]. The use of laboratory animals and of all procedures used in this study were approved by the Biomedical Ethics Committee of M.V. Lomonosov Moscow State University (Protocol number 149-g). Wistar rats (obtained from the Institute of General Pathology and Pathophysiology, Russia) were used. Rats were maintained at a controlled temperature and a 12/12 h light/dark cycle in the laboratory animal unit of the Biological Faculty, M.V. Lomonosov Moscow State University with access to food and water ad libitum. To obtain the offspring, sexually mature male and female rats were placed together for 4 days. Pregnant females were housed individually 3–4 days before the expected delivery. The next day after birth, the litters were reduced to 8 pups. Experiments were carried out on male rats aged 10 to 15 days (“young” in the following text) and 3 to 4 months (“adult” in the following text). In total, 30 adult and 75 young rats (offspring of 20 females) were used in the study. Rats were killed by decapitation, adult rats were first anesthetized by CO_2_.

The main object of the study was the saphenous artery. There are several reasons for this choice. The rat saphenous artery is a muscular-type artery, is densely innervated, and develops strong contractile responses to the stimulation of sympathetic nerves [21]. Therefore, along with its conduit function the saphenous artery participates in the control of cutaneous blood flow, which comprises up to 20% of the cardiac output in neonatal rats [22]. Importantly, according to our data, developmental alterations of several regulatory mechanisms observed in the saphenous artery are associated with corresponding changes at the level of blood pressure control [23]. Therefore, we consider the saphenous artery to be a representative artery from the peripheral circulation, i.e., the findings from the saphenous artery can be generalized to other vascular beds. In addition, the saphenous artery is large enough in 10–15-day-old rats to assess its responses by wire myography and to obtain a sufficient amount of tissue for qPCR, Western blot, and spectrophotometry analyses (described further).

### 2.2. Measurement of mRNA Expression Levels in Arterial Tissue by qPCR

Measurement of mRNA expression levels in arterial tissue by qPCR was performed as previously described [13,24]. Briefly, saphenous arteries were isolated, carefully cleaned from surrounding tissue and blood, and kept in RNAlater solution (Qiagen, Venlo, The Netherlands) at −20 °C pending further procedures. Each sample in the adult group included one artery and in the young group two arteries.

Arteries were homogenized in RLT lysis buffer (Qiagen, Venlo, The Netherlands) containing 1% β-mercaptoethanol and proteinase K (20 mg/mL, MP Biomedicals, Irvine, CA, USA). RNA was extracted using the ExtractRNA kit (cat. number BC032, Evrogen, Moscow, Russia) according to the manufacturer’s instructions and then processed with DNase I (cat. number EN0525, Fermentas, Waltham, MA, USA, 1000 U/mL). The RNA concentration was measured by a NanoDrop 1000 (Thermo Scientific, Waltham, MA, USA), and then all samples were diluted to an equal concentration of 35 ng/μL. Reverse transcription was performed using the MMLV RT kit (Evrogen, Moscow, Russia) according to the manufacturer’s manual. qPCR was run in the Bio-Rad CFX 96 Real-Time PCR System (Bio-Rad, Hercules, CA, USA), using qPCRmix-HS SYBR (Evrogen, Moscow, Russia). Primers used in this study were synthesized by Evrogen; their sequences are listed in Table 1. The mRNA expression level was calculated as E^(−Cq), where E is the primer efficiency, and Cq is the cycle number corresponding to the maximum of the second derivative of the fluorescence curve. Primer efficiency was identified using the LinRegPCR (version 2015.1) software [25]. All E values were close to 2.0. mRNA expression levels of investigated genes were normalized to the mRNA expression level of the housekeeping gene beta-actin (*Actb*), detected in the same sample. Importantly, mRNA expression values of *Actb* did not differ between the two age groups and were 2.2 × 10^−6^ ± 0.5 × 10^−6^ in adult arteries (n = 9) and 1.6 × 10^−6^ ± 0.3 × 10^−6^ in young arteries (n = 9, *p* > 0.05, unpaired Student’s *t*-test).

### 2.3. Measurement of Superoxide Dismutase and Catalase Activity in Arterial Tissue

Two saphenous arteries from one adult rat or four saphenous arteries from two young rats were used for one sample. Arteries were homogenized with ice cold PBS and centrifuged at 1500× *g* over 10 min at +4 °C. After that, probes were incubated with ice for 15 min. Supernatants were collected and frozen at −80 °C till further analysis.

SOD activity was determined by a spectrophotometric method based on the inhibition of the superoxide anion radical production rate by SOD, formed in the reaction of quercetin autooxidation in a slightly alkaline medium [26]. To determine the total SOD activity, 5 μL of the homogenate was mixed with 150 μL of a buffer containing 20 mM potassium phosphate buffer pH 7.8, 8 mM TEMED, and 0.08 mM EDTA in a 96-well plate. The reaction was started by adding 5 μL of 0.15 mg/mL quercetin in DMSO. To determine MnSOD activity, 3 mM KCN was added to the buffer. The change in optical density at 405 nm was recorded on a plate spectrophotometer INNO LTEK Co., Ltd. Instruments, Seongnam-si, Republic of Korea. One unit (U) of SOD activity was defined as the rate of 50% inhibition of the quercetin autooxidation reaction. Total SOD activity was expressed as U/mg total protein. Cu/ZnSOD activity was calculated as the difference between total SOD and MnSOD activities. Total protein content was determined by the modified Lowry method [27].

Catalase activity was determined spectrophotometrically using a method based on the reaction of excess (unreacted) hydrogen peroxide with FOX reagent [28]. The substrate–buffer mixture for catalase contained 50 mM potassium phosphate buffer (pH 7.4) and 0.2 mM hydrogen peroxide. In a 96-well plate, the supernatant of vascular homogenates was mixed with the substrate–buffer mixture at a ratio of 1:20 (*v*/*v*) and incubated for 10 min at room temperature. The reaction was stopped by the addition of FOX reagent and further incubated for 30 min at room temperature until a stable violet coloration developed. Optical density was recorded at 560 nm using a microplate spectrophotometer (INNO LTEK Co., Ltd. Instruments, Seongnam-si, Republic of Korea). The concentration of decomposed hydrogen peroxide was calculated using the millimolar extinction coefficient of 235 mM^−1^·cm^−1^. One unit (U) of catalase activity was defined as the amount of enzyme required to decompose 1 μmol of hydrogen peroxide per minute. Catalase activity in vascular homogenates was expressed as U/mg of total protein. Total protein concentration was determined using the modified Lowry method [27].

### 2.4. Measurement of Protein Abundance in Arterial Tissue by Western Blotting

Measurement of protein abundance in arterial tissue was performed as described previously [13,29]. Four saphenous arteries from two young rats or two saphenous arteries from one adult rat were used for one sample. The endothelium was rapidly removed after isolation of the artery with the use of a custom-made wire myograph analogue [30]. Thereafter, saphenous arteries were quickly frozen in liquid nitrogen and kept at −80 °C until further analysis. Samples were homogenized in ice-cold SDS buffer supplemented with protease inhibitor cocktail (Roche, Bazel, Switzerland) and phosphatase inhibitors (2 mg/mL NaF and 180 mg/mL Na_3_VO_4_), centrifuged at 16,900× *g* for 5 min; the supernatant was kept at −20 °C till further analysis. Proteins were separated by SDS-PAGE and transferred to nitrocellulose membranes (Santa Cruz, Dallas, TX, USA) using the Trans-Blot Turbo transfer system (Bio-Rad). The transfer was visualized with Ponceau S staining. Thereafter, membranes (each containing samples from adult and young groups) were cut into two pieces at the level of appr. 28 kDa based on the corresponding levels of protein markers (Abcam, Cambridge, UK, cat. number 116027). The membranes were blocked with 5% nonfat milk (AppliChem, Darmstadt, Germany) in TBS with 0.1% Tween 20 (TBSt). Then, two pieces of the first membrane were incubated overnight with antibodies against GAPDH (mouse, Abcam, cat. number 125247, 1:2000 in TBSt with 5% milk, molecular weight 37 kDa) or SOD1 (rabbit, Sigma, St. Louis, MO, USA, cat. number SAB5200083 1:4000 in TBSt with 5% milk, molecular weight 23 kDa), and one piece of the second membrane was incubated overnight with antibodies against SOD3 (rabbit, Enzo, New York, NY, USA, cat. number ADI-SOD-106-F 1:1000 in TBSt with 5% milk, molecular weight 35–40 kDa). The next day, the membranes were washed from the primary antibodies and incubated with appropriate secondary antibodies: anti-mouse (Cell Signaling, Boston, MA, USA, cat. number 7076, 1:5000 in 5% milk) or anti-rabbit (Cell Signaling, Boston, MA, USA, cat. number 7074, 1:10,000 in 5% milk) for 1 h and visualized with SuperSignal West Dura Substrate (Thermo Fisher Scientific, Waltham, MA, USA) using a ChemiDoc (Bio-Rad, Hercules, CA, USA). Of note, since SOD3 and GAPDH have similar molecular weights (close to 40 kDa), membrane stained with antibodies against SOD3 was stripped using stripping buffer (for composition see Section 2.6), blocked again as described above, and incubated overnight with antibodies against GAPDH. Western blotting experiments were analyzed using the Image Lab software 6.0 (Bio-Rad, Hercules, CA, USA). SOD1 and SOD3 protein abundance was normalized to GAPDH in each sample—the rationale for using GAPDH as a reference protein for comparing arteries from adult and early postnatal animals was provided previously [31])—and then the average ratio of the «Adult» group was taken as 100%.

### 2.5. Wire Myograph Experiments

Saphenous arteries were carefully cleaned from surrounding tissue in PSS for vessel dissection (PSS I), cut into 2-mm-long segments, and mounted in a wire myograph (410 A or 620 M, DMT A/S, Aarhus, Denmark) to measure the isometric force. Experiments were carried out both on arteries with an intact endothelium and on arteries with the endothelium removed. The endothelium was removed mechanically using a rat whisker by performing rotational movements inside the artery.

During the subsequent experiments, arterial segments were kept in PSS II. The myograph chambers were heated to 37 °C and continuously bubbled with 5% CO_2_ in O_2_ to maintain pH at 7.4. Data were recorded at 10 Hz using an analogue-to-digital converter (E14-140M, L-CARD, Moscow, Russia) and the PowerGraph 3.3 software (DISoft, Moscow, Russia). Each arterial segment was stretched to 0.9 × d100 (90% of the inner diameter it would have at a transmural pressure of 100 mmHg), corresponding to maximum active force development [13,32].

At the beginning of each experiment, all vessels were exposed to a standard start-up procedure. This included the following: (a) application of noradrenaline (10 μM); (b) application of acetylcholine (10 μM) on the top of methoxamine-induced contraction (1 μM) to confirm the integrity or removal of the endothelium (by the presence or the absence of a relaxation response, respectively); (c) application of methoxamine (10 μM).

Two experimental protocols were used in the study. The first experimental protocol included two sequential concentration–response relationships to methoxamine (concentration range from 0.01 to 100 μM, for three minutes each). The first relationship was started 20 min after the end of the activation procedure. After washout, one arterial segment was incubated for 20 min with one of the following substances or their combinations: inhibitor of Cu/ZnSOD DETC (1 mM), NO-synthase inhibitor L-NNA (0.1 mM), soluble guanylate cyclase inhibitor ODQ (1 µM), NO-donor sodium nitroprusside SNP (0.1 µM), superoxide dismutase SOD (300 U/mL), catalase (1500 U/mL). The other segment was incubated with an equivalent volume of the solvent (DMSO or H_2_O or their combination). Then, the second concentration–response relationship was obtained. The second experimental protocol also included sequential concentration–response relationships. The first one was to methoxamine (concentration range from 0.01 to 100 μM), which allowed us to determine the concentration of methoxamine that causes a contraction of approximately 60% of the maximum. After washout, contractions at the level of approximately 60% of the maximum in two arterial segments were induced, and then H_2_O_2_ (concentration range from 1 μM to 0.3 mM, for 2 min each) was added sequentially to one of the segments and an equivalent volume of H_2_O was added to another segment (Time-control).

To calculate active force values at each methoxamine concentration, the force value at the fully relaxed state was subtracted from all recorded values. All active force values obtained during the second concentration–response relationship to methoxamine were expressed as the percentage of the maximum active force developed during the respective first concentration–response relationship or as a percentage of the precontraction level (for H_2_O_2_). Areas under the curves (AUCs) were calculated in several experimental series as an additional way to evaluate the effects of inhibitors (Table S1). The internal diameter (d100), maximal force and values of acetylcholine-induced relaxation of the arteries in different series of wire-myography experiments are presented in Table S2.

### 2.6. Solutions

1. PSS for vessel isolation (PSS I), in mM: 145 NaCl, 4.5 KCl, 1.2 NaH_2_PO_4_, 1 MgSO_4_, 0.1 CaCl_2_, 0.025 EDTA, 5 HEPES (pH 7.4).

2. PSS for myograph experiments (PSS II), in mM: 120 NaCl, 26 NaHCO_3_, 4.5 KCl, 1.2 NaH_2_PO_4_, 1.0 MgSO_4_, 1.6 CaCl_2_, 5.5 D-glucose, 0.025 EDTA, 5 HEPES; equilibrated with 5% CO_2_ in 95% O_2_ (pH 7.4).

3. SDS-buffer: 0.0625 M Tris-HCl (pH 6.8), 2.5% SDS, 10% water-free glycerin, 2.47% dithiothreitol, 0.002% bromophenol blue.

4. TBS: 50 mM Tris-HCl, 150 mM NaCl, pH 7.6.

5. TBSt: TBS with 0.1% Tween.

6. Stripping buffer: 0.2 M glycine, 0.1% SDS, 1% Tween (pH 2.2).

### 2.7. Drugs

Noradrenaline, acetylcholine, methoxamine, DETC, catalase (all dissolved in H_2_O) and ODQ (dissolved in DMSO) were obtained from Sigma (St. Louis, MO, USA). L-NNA (dissolved in H_2_O) was obtained from Alexis Biochemicals (San Diego, CA, USA). SOD (dissolved in H_2_O) was obtained from BiosynthCarbosynth (Staad, Switzerland).

### 2.8. Statistics

Statistical analysis was performed using GraphPad Prism 8.0. The normality of data distribution was checked using the Shapiro–Wilk test; data are presented as the mean and standard error of the mean or as the median and interquartile range, depending on the type of data distribution. Statistical analysis was performed using two-way repeated measures ANOVA, one-way ANOVA, the parametric Student’s *t*-test, and the Mann–Whitney test. Differences were accepted as statistically significant if the *p* value was less than 0.05; n represents the number of animals, i.e., biological replicates.

## 3. Results

### 3.1. mRNA Expression of SOD Isoforms in Saphenous Arteries in Adult and Young Rats

**Figure 1:**
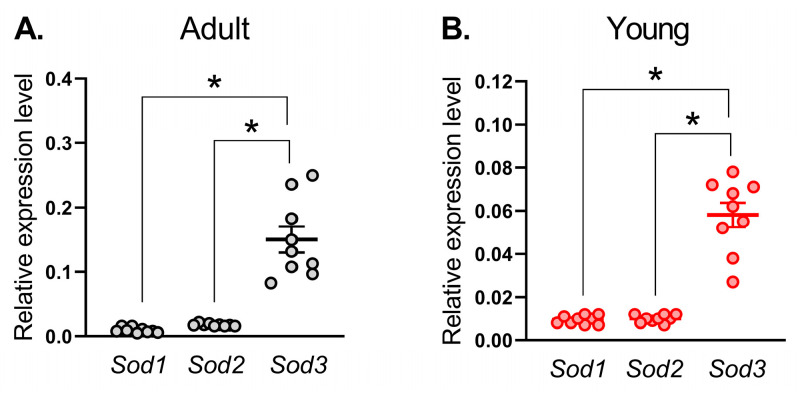
mRNA expression of superoxide dismutase isoforms in saphenous arteries of adult (**A**) and young (**B**) rats. Data are normalized to the beta-actin (*Actb*) expression level. The number of samples in each group is 9. * *p* < 0.05 (one-way ANOVA with Tukey’s multiple comparisons test).

First, we identified the expression profile of SOD isoforms in the saphenous artery of young and adult rats. For this purpose, we compared mRNA contents of *Sod1*, *Sod2*, and *Sod3* genes. mRNAs of all three isoforms were detected in arterial tissue from adult and young rats (Figure 1). Among these three isoforms, mRNA of *Sod3* was most abundant in both adult and young saphenous arteries (Figure 1).

### 3.2. Activity of SODs and Catalase in Saphenous Arteries in Adult and Young Rats

**Figure 2:**
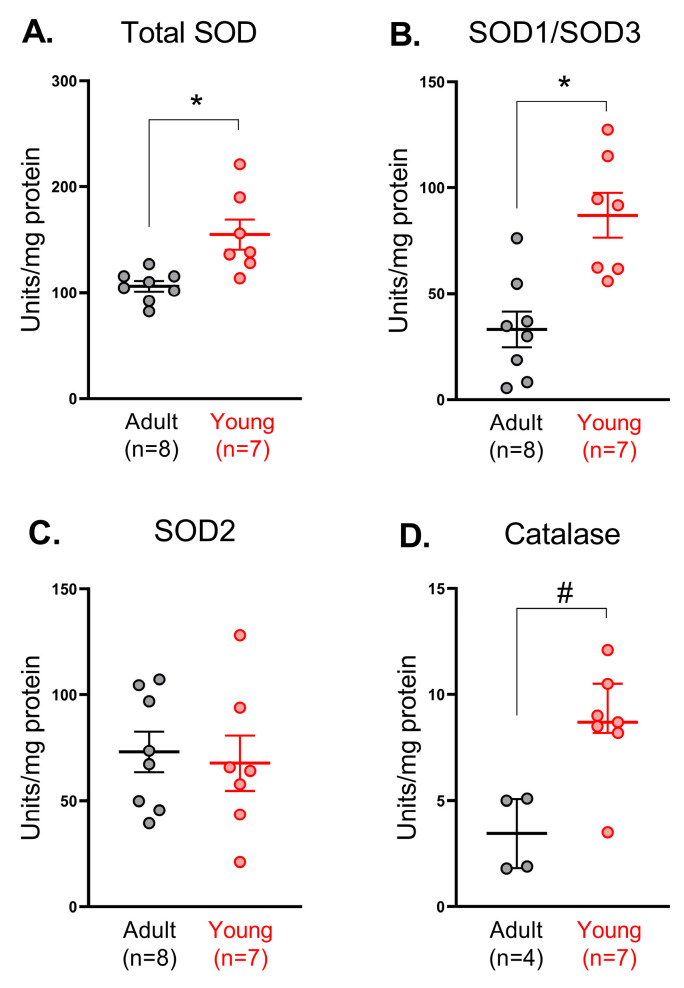
Activity of superoxide dismutases (total (**A**), Cu/ZnSOD (**B**) or MnSOD (**C**)) and catalase (**D**) in saphenous arteries of adult and young rats. Data are normalized to the total protein amount in the same sample; n is the number of animals. * *p* < 0.05 (unpaired Student’s *t*-test), # *p* < 0.05 (Mann–Whitney test).

Next, we evaluated the activity of SODs in saphenous arteries from adult and young rats. Total SOD activity was higher in arterial homogenates from young in comparison to adult rats (Figure 2A), which, apparently, is due to the higher activity of Cu/ZnSOD isoforms (SOD1 and SOD3) (Figure 2B), but not MnSOD (SOD2) (Figure 2C). Catalase activity was higher in arterial homogenates from young in comparison to adult rats (Figure 2D).

### 3.3. SOD1 and SOD3 Protein Abundances in Saphenous Arteries in Adult and Young Rats

**Figure 3:**
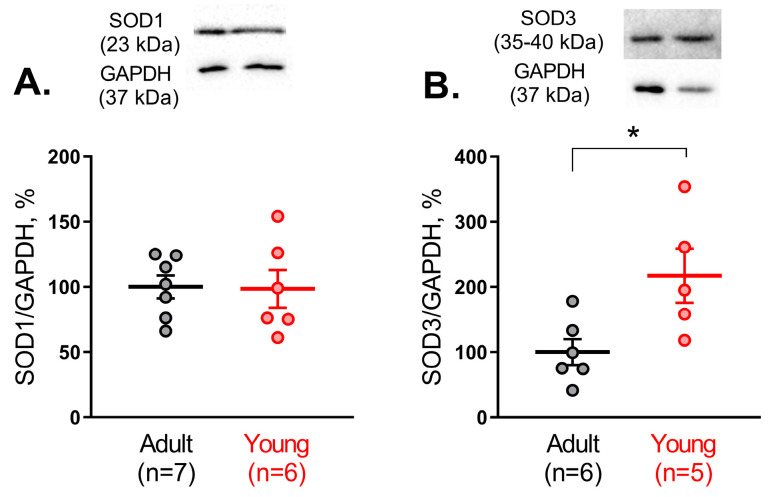
Protein abundance of SOD1 (**A**) and SOD3 (**B**) in saphenous arteries of adult and young rats. Data are normalized to GAPDH. The mean value of SOD1 or SOD3 protein content in the adult group is taken as 100%; n is the number of animals. * *p* < 0.05 (unpaired Student’s *t*-test). The whole-length Western blot membranes are shown in Supplemetary Materials (Figure S1).

Since Cu/ZnSODs demonstrated higher activity in arteries from young rats, we further compared the protein abundance of SOD1 and SOD3 between arteries of young and adult rats. We did not detect any difference in the protein amount of SOD1 in arterial tissue of adult and young rats (Figure 3A), while the protein amount of SOD3 was augmented in young rats (Figure 3B).

### 3.4. Effect of H_2_O_2_ on Arterial Tone in Saphenous Arteries of Young Rats

One of the main functions of SODs in the cells is the catalysis of O_2_^•−^ to H_2_O_2_, which in its turn has a number of signaling functions, including vasomotor effects [1]. However, the vasomotor effect of H_2_O_2_ has been much less studied in arterial smooth muscle at early postnatal age. Therefore, we evaluated the effects of H_2_O_2_ on the tone of endothelium-denuded saphenous arteries of young rats.

**Figure 4:**
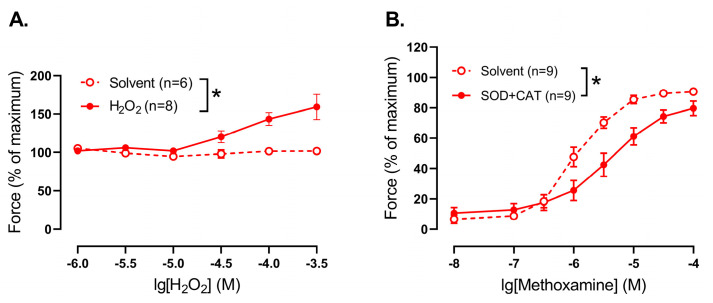
Effect of H_2_O_2_ addition and elimination on the tone of endothelium-denuded saphenous arteries from young rats. (**A**) Concentration–response relationship of precontracted by methoxamine arteries to exogenous H_2_O_2_. (**B**) Contractile responses to methoxamine in the presence of solvent or superoxide dismutase (SOD, 300 U/mL) together with catalase (CAT, 1500 U/mL); n is the number of animals. * *p* < 0.05 (two-way repeated measures ANOVA).

First, we added H_2_O_2_ to precontracted (approximately 60% of maximal force) endothelium-denuded segments of saphenous arteries of young rats. In the concentration range used H_2_O_2_ caused additional contraction of the arteries in comparison to vehicle application (Figure 4A). Importantly, the initial values of precontraction did not differ between solvent- and H_2_O_2_-treated arteries and were 57 ± 7% and 64 ± 4% of maximal force, respectively, (*p* > 0.05, unpaired Student’s *t*-test).

Second, when H_2_O_2_ was eliminated from the extracellular space by addition of superoxide dismutase (300 U/mL) together with catalase (1500 U/mL, promotes H_2_O_2_ breakdown), methoxamine-induced contractile responses of young saphenous arteries were reduced compared to untreated vessels (Figure 4B).

### 3.5. Effect of a Cu/ZnSOD Inhibitor on Arterial Contractile Responses in Saphenous Arteries of Adult and Young Rats

In the following experiments we focused on SOD1 and SOD3 since these superoxide dismutase isoforms (i) are abundant in the saphenous arteries in both age groups; (ii) demonstrate higher activity and amount in arteries of young rats, and (iii) can be blocked by the inhibitor DETC (unlike SOD2) [7], which allowed us to evaluate their role at the functional level, i.e., in the regulation of contractile responses of isolated arterial segments.

**Figure 5:**
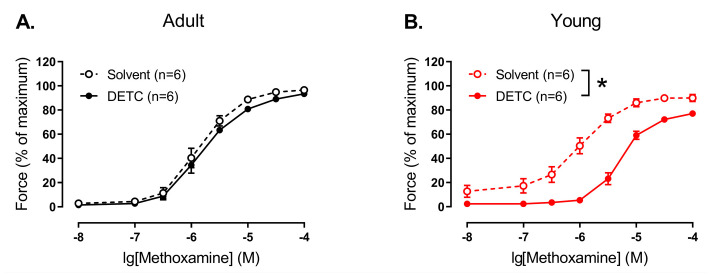
Effect of the Cu/ZnSOD inhibitor DETC on the contractile responses to methoxamine of endothelium-denuded arteries from young and adult rats. Concentration–response relationships to methoxamine in the presence of solvent (H_2_O) or DETC (1 mM) of endothelium-denuded arteries from adult (**A**) and young (**B**) rats; n is the number of animals. * *p* < 0.05 (two-way repeated measures ANOVA).

We did not detect any effect of pre-treatment with DETC (1 mM) on methoxamine-induced contractile responses in endothelium-denuded arteries of adult rats (Figure 5A). However, DETC considerably reduced contractile responses to methoxamine of endothelium-denuded arteries of young rats (Figure 5B).

**Figure 6:**
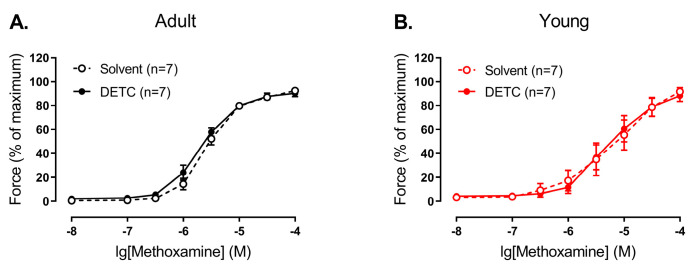
Effect of the Cu/ZnSOD inhibitor DETC on the contractile responses to methoxamine of endothelium-intact arteries from young and adult rats. Concentration–response relationships to methoxamine in the presence of solvent (H_2_O) or DETC (1 mM) of endothelium-intact arteries from adult (**A**) and young (**B**) rats; n is the number of animals.

In contrast, we did not detect any effect of DETC in endothelium-intact arteries of both adult and young rats (Figure 6).

### 3.6. Effect of a Cu/ZnSOD Inhibitor on Arterial Contractile Responses in Saphenous Arteries of Young Rats During Manipulation of NO-Dependent Signaling

Next, we interrogated which endothelium-dependent pathway counteracts the functional impact of SOD1 and SOD3 in smooth muscle of saphenous arteries of young rats. Since the anticontractile effect of endothelial NO is particularly pronounced during early postnatal ontogenesis [33], we focused primarily on the NO pathway.

**Figure 7:**
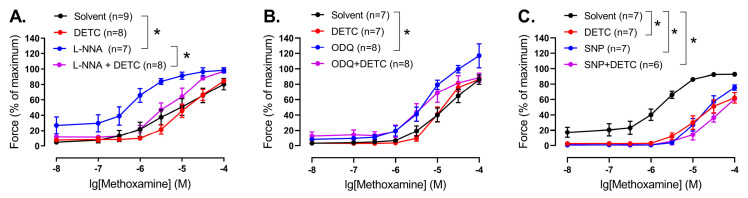
Effect of the Cu/ZnSOD inhibitor DETC on the contractile responses to methoxamine of arteries from young rats during manipulation of NO-dependent signaling. (**A**) Concentration–response relationships to methoxamine in the presence of solvent (H_2_O) or DETC (1 mM), or the NO-synthase inhibitor L-NNA (100 µM), or L-NNA together with DETC of endothelium-intact saphenous arteries. (**B**) Concentration–response relationships to methoxamine in the presence of solvent (H_2_O) or DETC (1 mM), or the soluble guanylate cyclase inhibitor ODQ (1 µM), or ODQ together with DETC of endothelium-intact saphenous arteries. (**C**) Concentration–response relationships to methoxamine in the presence of solvent (H_2_O), or DETC (1 mM), or the NO donor sodium nitroprusside SNP (0.1 µM), or SNP together with DETC of endothelium-denuded saphenous arteries; n is the number of animals. * *p* < 0.05 (two-way repeated measures ANOVA, followed with Tukey’s multiple comparisons test).

Thus, we evaluated the effect of DETC in the presence of the NO-synthase inhibitor L-NNA. L-NNA increased contractile responses of endothelium-intact arteries of young rats (Figure 7A, Table S1). Importantly, DETC decreased contractile responses of the arteries in the presence of L-NNA (Figure 7A, Table S1).

Next, we evaluated the effect of DETC in the presence of ODQ, an inhibitor of soluble guanylate cyclase, the intracellular target of NO. ODQ caused an increase in the contractile responses of endothelium-intact arteries of young rats (Figure 7B, Table S1). At the same time, we did not detect any effect of DETC in the presence of ODQ (Figure 7B, Table S1).

In order to test whether NO (or its derivatives) may neutralize SODs directly, we assessed the effects of DETC in endothelium-denuded arteries in the presence of the NO donor sodium nitroprusside (SNP). SNP attenuated arterial contractile responses (Figure 7C, Table S1). Importantly, we did not detect any effect of DETC in the presence of SNP (Figure 7C, Table S1).

## 4. Discussion

In the present study, for the first time, age-related changes of SOD1 and SOD3 vasomotor functional contribution in systemic arteries were evaluated. We showed that activities of Cu/ZnSODs (SOD1 and SOD3) as well as SOD3 protein content were considerably increased in arterial tissue from early postnatal rats in comparison to adult animals. In accordance with these results, functional experiments indicate that SOD1 and SOD3 functioning promotes contraction of arterial smooth muscle of young, but not adult rats. In addition, endothelial NO (or its derivatives) considerably counteracts the SOD1 and SOD3 influence in arterial smooth muscle of early postnatal rats.

Age-related changes in SOD expression and/or activity were addressed previously, but only in the pulmonary circulation, not in the systemic arteries. For example, the activity and protein amount of Cu/ZnSOD were significantly decreased in the lung tissue of newborn rabbits compared to adults [15]. Similarly, the expression of Cu/ZnSOD mRNA in human lung tissue increased from newborns to adults [16]. In addition, the protein content of SOD1 and SOD3 in the lung tissue of adult mice was higher than in 7-day-old mice [17]. Of note, lung tissue, used in the cited studies, includes many types of cells, not just vascular ones. Nevertheless, taken together, these data suggest that the activity and expression of SOD1 and SOD3 in the pulmonary circulation at the early stages of postnatal development is decreased, making newborn organisms more susceptible to persistent pulmonary hypertension development [18]. Our study, for the first time, addresses the expression pattern and age-related changes in SOD activity/protein content in systemic arteries. We found that the mRNA expression level of *Sod3* was the highest in both adult and young rats. At the same time, total SOD activity as well as Cu/ZnSOD activity was considerably increased in arterial tissue from early postnatal rats in comparison to adult. Importantly, although SOD1 protein content did not differ between arterial smooth muscle of adult and young rats, the protein amount of SOD3 in the arterial smooth muscle of 10–15-day old rats was increased in comparison to adults. Thus, in contrast to the pulmonary circulation, arteries of the systemic circulation demonstrate a higher activity and protein amount of Cu/ZnSODs during the early postnatal period.

As mentioned, SODs catalyze the conversion of O_2_^•−^ into H_2_O_2_, which participates in cell signaling. H_2_O_2_ may cause both relaxation and contraction of arteries, as it was shown for different arteries from adult animals [2,3,4,5,6]. However, much less is known about vasomotor effects of H_2_O_2_ in arteries of newborn/early postnatal organisms. A few studies demonstrated transient H_2_O_2_-induced contractions of piglets’ pial arterioles [34] as well as small pulmonary arteries of 1–2-week-old piglets [35]. In accordance with this, our data demonstrate that H_2_O_2_ addition promotes contraction, while H_2_O_2_ elimination, on the contrary, results in the weakening of contractile responses of endothelium-denuded saphenous arteries from 10–15 day-old rats, indicating a pro-contractile influence of H_2_O_2_ in arterial smooth muscle in the early postnatal period. Of note, previously we demonstrated that H_2_O_2_ elimination had no effect on methoxamine-induced contractile responses of saphenous arteries of adult rats [33], indicating that H_2_O_2_ does not have a pronounced vasomotor effect in this artery at adulthood.

Our next step was to evaluate the functional impact of H_2_O_2_, produced by Cu/ZnSODs, on the regulation of smooth muscle contraction in saphenous arteries from two age groups of rats. To do so, we used DETC, which chelates Cu^2+^, leading to a decrease of enzyme activity [7] and, consequently, H_2_O_2_ content in the arterial wall [36]. In accordance with our molecular experiments, which demonstrated low activity and protein amount of Cu/ZnSODs in the arteries of adult rats, DETC had no influence on methoxamine-induced contractions of endothelium-denuded arteries in adult animals. Similarly, in previous studies, DETC had no effect on phenylephrine-induced contractile responses of rat aorta [8,9] and contractile responses to PGF2alpha of mouse aorta [12]. Along with that, DETC augmented vasocontraction of mouse aorta in response to serotonin [12] and norepinephrine [10] as well as induced further vasoconstriction of precontracted by norepinephrine rat mesenteric arteries [11], pointing to an anti-contractile influence of H_2_O_2_, produced by Cu/ZnSODs. Thus, the vasomotor contribution of SODs may depend on the artery type and/or the agonist used. Importantly, our data demonstrate that the vasomotor influence of SODs may also depend on the stage of the organism’s development: DETC led to pronounced weakening of methoxamine-induced contraction of endothelium-denuded saphenous arteries of young rats. In accordance with the described above pro-contractile influence of H_2_O_2_ in endothelium-denuded saphenous arteries of young rats, the weakening of arterial contraction after Cu/ZnSOD inhibition indicates a pro-contractile role of H_2_O_2_, produced by these enzymes, in the smooth muscle of early postnatal saphenous arteries. Of note, the mechanism of such pro-contractile influence remains unclear. Previously we demonstrated, that ROS, produced by NADPH-oxidase, promote vasocontraction of saphenous artery in early postnatal rats by activation of LTCC [24]. Taking into account (1) the high production of O_2_^•−^ (demonstrated previously [13]) and (2) high activity of Cu/ZnSODs in saphenous arteries of early postnatal rats (demonstrated in the present study), it is possible that H_2_O_2_ formed from O_2_^•−^ activates LTCC. However, such a hypothesis should be tested in future studies.

It should be also noted that the activity of one of the H_2_O_2_ scavengers, catalase, was higher in arteries of young compared to adult rats. As already mentioned, we demonstrated previously increased O_2_^•−^ production [13] in saphenous arteries of early postnatal rats. Therefore, apparently, saphenous arteries of early postnatal rats exhibit both higher levels of ROS production and ROS degradation. Such an augmentation of catalase activity in young arteries may serve as an adjustment for increased ROS levels, where initially increased production leads to a rise in ROS levels and a secondary increase in ROS degradation then establishes a new, higher ROS steady state with balanced production and degradation. However, we do not know the kinetics of these processes and cannot say whether such a compensation occurs to the same extent as in adults. Determination of the kinetics of the production and degradation processes requires a large number of experiments that were beyond the scope of the present study. In addition, a set of data demonstrating (1) higher ROS production (O_2_^•−^ [13]), (2) higher Cu/ZnSODs activity, (3) higher SOD3 protein content in saphenous arteries of early postnatal rats allows the suggestion that the differences in functional impact of Cu/ZnSODs between young and adult arteries are mainly associated with ROS generation than with their downstream signaling.

Importantly, vasomotor effects of many molecules, including H_2_O_2_, on smooth muscle tone may depend on the endothelium. For example, endothelium denudation eliminated contraction of pressurized gracilis muscle arterioles of adult rats in response to 0.1 mM of H_2_O_2_ [3]. In addition, incubation with SOD weakened phenylephrine-induced contraction of endothelium-intact pulmonary arteries of newborn rabbits, which was not observed after endothelium denudation [37]. Therefore, we evaluated the effects of the Cu/ZnSOD inhibitor DETC in endothelium-intact saphenous arteries. The presence of endothelium did not affect the effect of DETC in adult arteries: contractile responses to methoxamine were not affected by DETC. However, DETC did not weaken anymore the contraction of young arteries when the endothelium was left intact. Thus, endothelium is able to counteract the vasomotor impact of Cu/ZnSODs in the arteries of young rats. Since the anti-contractile effect of endothelial NO is particularly pronounced during early postnatal ontogenesis [33], we hypothesized that the lack of DETC effect in endothelium-intact arteries of young rats is primarily associated with the NO pathway. Indeed, DETC decreased contractile responses of young arteries in the presence of the NO-synthase inhibitor L-NNA, as we previously observed in vessels without endothelium (Figure 5B). This indicates that endothelial NO counteracts the vasomotor impact of Cu/ZnSODs in the arterial smooth muscle of young rats.

Further experiments were aimed to answer the question of how NO counteracts the effect of DETC in the arteries of young rats. According to the literature, the activity of Cu/ZnSODs can be altered by post-translational modifications via phosphorylation [38]. Therefore, we suggested that the signaling cascade activated by NO in the smooth muscle cell (activation of soluble guanylate cyclase followed by activation of PKG) may be involved. To answer this question, we evaluated the effect of DETC in the presence of the soluble guanylate cyclase inhibitor ODQ. Importantly, the effectiveness of using 1 µM of ODQ in the saphenous artery of rats was previously demonstrated [33]. Our data show that DETC had no effect in the presence of ODQ, indicating that the neutralizing effect of NO on the effects of DETC in endothelium-intact arteries of young rats is not linked with soluble guanylate cyclase activation, and, probably, its main target in smooth muscle cell—PKG. Of note, Cu/ZnSODs may be nitrosylated, which may also change their activity [38]. Therefore, we suggested that NO (or its derivatives, such as peroxynitrite (ONOO^−^)) may neutralize SODs directly. To check this, we assessed the effects of DETC in endothelium-denuded arteries in the presence of the NO donor sodium nitroprusside (SNP), which mimics best the effect of naturally produced endothelial NO [39]. Importantly, incubation with DETC did not further attenuate the contractile response in the presence of SNP, similar to that which we observed in endothelium-intact arteries. Thus, NO (or its derivatives, such as peroxynitrite (ONOO^−^)) counteracts the SOD1 and SOD3 functional impact in the arterial smooth muscle of young rats regardless of the classical signaling cascade of NO, in particular soluble guanylate cyclase activation.

### Limitations

The present study has a number of limitations. First, the effect of exogenous H_2_O_2_ was not studied in the arteries of adult rats. Second, sex-dependent differences were not assessed, since only males were used. Third, H_2_O_2_ downstream signaling mechanisms were not explored.

## 5. Conclusions

In conclusion, our novel findings demonstrate that Cu/ZnSODs promote contraction of saphenous arteries, an artery of the systemic circulation, at the early postnatal period, but not at adult age. Moreover, endothelial NO, the anti-contractile influence of which is particularly high at the early stages of postnatal development, counteracts the pro-contractile influence of Cu/ZnSODs in early postnatal, but not in adult arteries. The data obtained should be taken into account at least for several reasons. First, our data complement and develop knowledge about the altered vasomotor contributions of ROS in early postnatal ontogenesis. In addition, the more pronounced functional contribution of Cu/ZnSODs, along with the previously demonstrated high vasomotor influence of NADPH oxidases [13,24], in arteries of young animals may be associated with the stimulation of smooth muscle differentiation [40,41] and angiogenesis [42] by ROS, which actively occur in the early postnatal period. Second, the direction of age-related changes in the functional contribution of Cu/ZnSODs in the systemic circulation appears to be opposite to that in the pulmonary circulation, as described in the literature. Therefore, we have received further evidence for the differences in the functioning between the systemic and pulmonary circulations. Finally, the data obtained need to be taken into account when developing therapeutic approaches in newborns, including those intended to treat “oxygen radical disease in the newborn” [18,43,44].

## Figures and Tables

**Table 1:** Gene specific primers used in the study.

Gene Name	Gene Accession Numbers	Forward	Reverse
*Sod1*	NM_017050.1	GTACCACTGCAGGACCTCAT	CCACCTTTGCCCAAGTCATC
*Sod2*	NM_017051.2	CCGTGGTGGGTGTTTTGTAG	CGTCCAAGCAATTCAAGCCT
*Sod3*	NM_012880.2	CGCCTCCAGTCATCCTAGAG	AAAGTGTCCTGGTCTCCGAG
*Actb*	NM_031144.3	CAGGGTGTGATGGTGGGTATGG	AGTTGGTGACAATGCCGTGTTC

## Data Availability

All data generated during this study are available from the corresponding author on reasonable request.

## Supplementary Materials

The following supporting information can be downloaded at: https://www.mdpi.com/article/10.3390/antiox14101231/s1. Table S1: Area under the curve (AUC) in experimental series on evaluation of the Cu/ZnSOD inhibitor effects on the contractile responses to methoxamine of arteries from young rats during manipulation of NO-dependent signaling. Table S2: The internal diameter (d100), maximal force, and values of acetylcholine-induced relaxation of the arteries in different series of wire-myography experiments. Figure S1: Original unprocessed images of Western blot membranes.

## Abbreviations

The following abbreviations are used in this manuscript:

ROS	Reactive oxygen species
SOD	Superoxide dismutase
DETC	Diethyldithiocarbamate
NO	Nitric oxide
SNP	Sodium nitroprusside
