# Supplementary material for: The Vasomotor Impact of Cu/ZnSODs Is Higher in Arterial Smooth Muscle of Early Postnatal Rats Compared to Adult Animals
Source: Antioxidants (Basel). 2025 Oct 14;14(10):1231. doi: 10.3390/antiox14101231 (PMC12561006; doi:10.3390/antiox14101231)
Supplement: Supplementary file 1 [file antioxidants-14-01231-s001.zip › Figure S1. WB images original SOD1 SOD3.pdf]

Original unprocessed images of Western blot membranes used in the article

Membrane 1

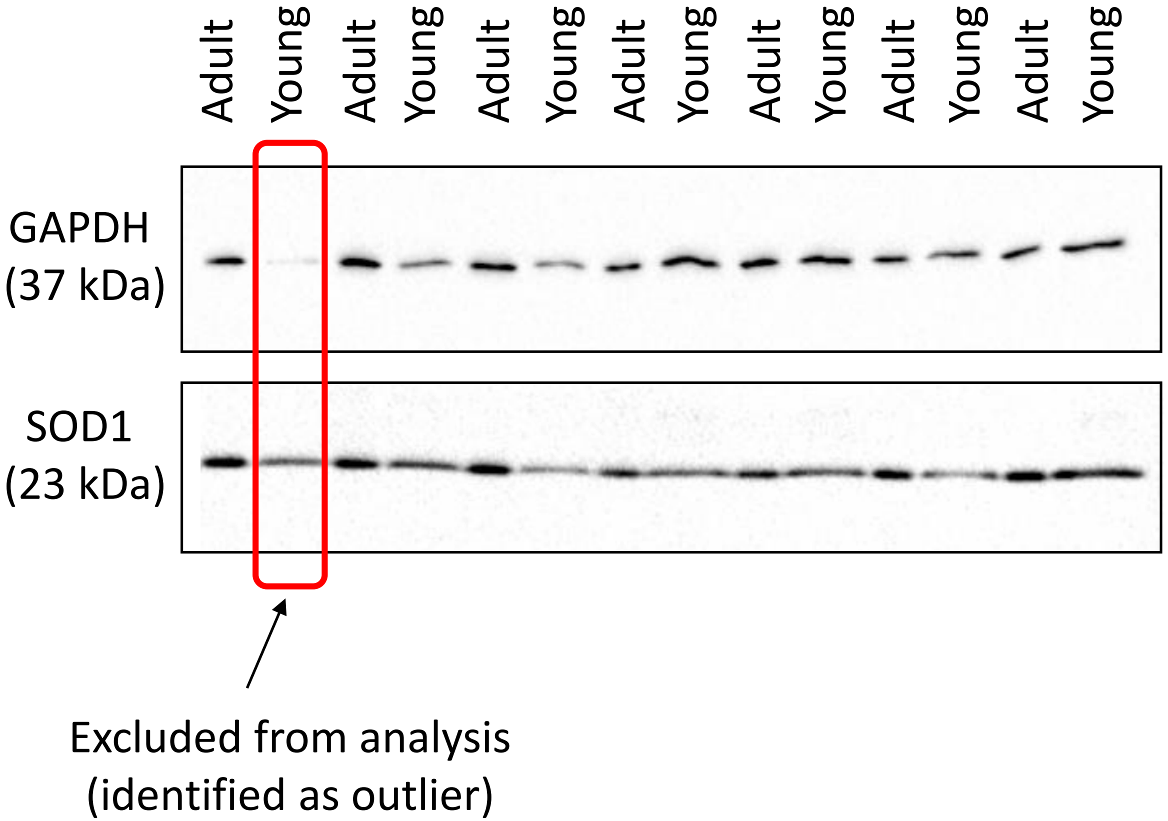

Membrane 2

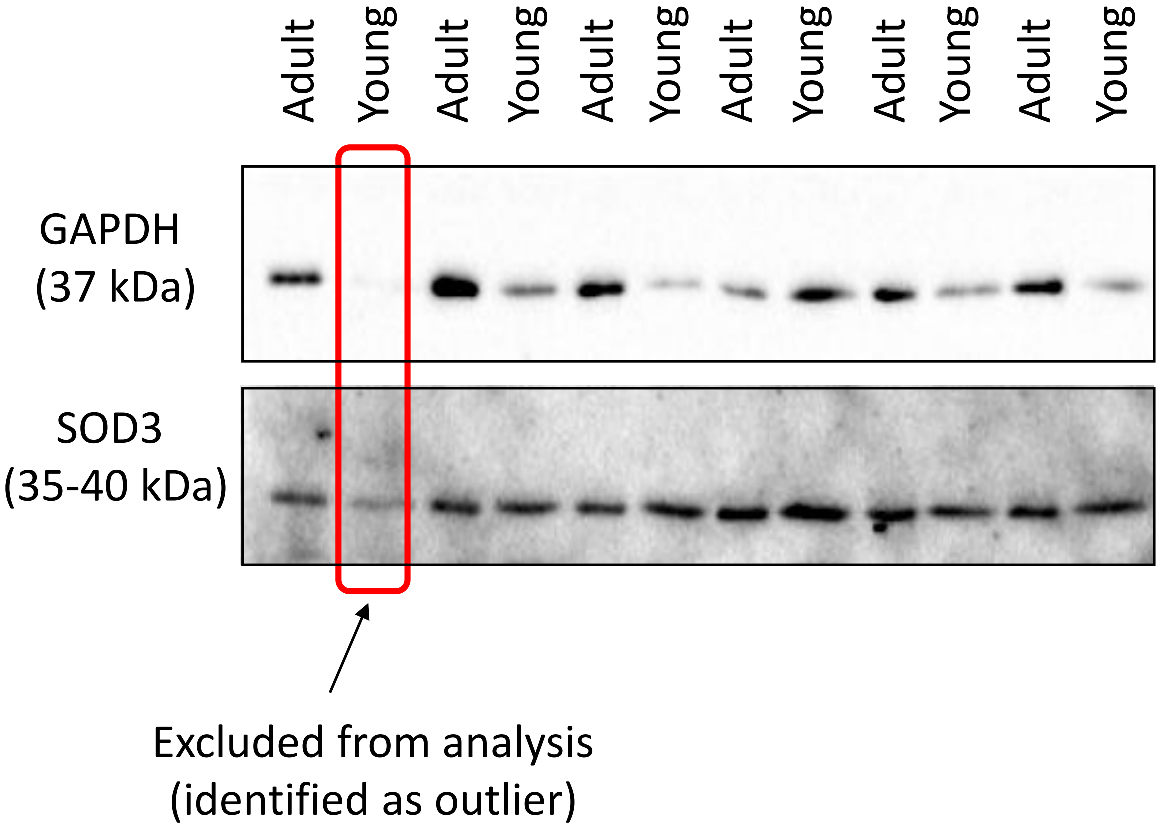

Molecular markers are not visible because they do not fluoresce
