# Supplementary material for: The Vasomotor Impact of Cu/ZnSODs Is Higher in Arterial Smooth Muscle of Early Postnatal Rats Compared to Adult Animals
Source: Antioxidants (Basel). 2025 Oct 14;14(10):1231. doi: 10.3390/antiox14101231 (PMC12561006; doi:10.3390/antiox14101231)
Supplement: Supplementary file 1 [file antioxidants-14-01231-s001.zip › Supplementary Tables.pdf]

Table S1. Area under the curve (AUC) in experimental series on evaluation of the Cu/ZnSOD inhibitor effects on the contractile responses to methoxamine of arteries from young rats during manipulation of NO-dependent signaling

	AUC, a.u.
Series of experiment (Figure 7A)	
Solvent (n=9)	112±25
DETC (n=8)	107±14
L-NNA (n=7)	248±27$
L-NNA+DETC (n=9)	156±16&
Series of experiment (Figure 7B)	
Solvent (n=7)	94±12
DETC (n=7)	92±11
ODQ (n=8)	166±17$
ODQ+DETC (n=8)	152±27
Series of experiment (Figure 7C)	
Solvent (n=7)	201±20
DETC (n=7)	68±13@
SNP (n=7)	64±9$
SNP+DETC (n=6)	44±10

@ - between Solvent and DETC (One-Way ANOVA with Holm-Šídák's multiple comparisons test); $ - between Solvent and L-NNA or Solvent and ODQ or Solvent and SNP (One-Way ANOVA with Holm-Šídák's multiple comparisons test); & - between L-NNA and L-NNA+DETC (One-Way ANOVA with Holm-Šídák's multiple comparisons test).

Table S2. The internal diameter (d_{100}), maximal force and values of acetylcholine-induced relaxation of the arteries in different series of wire-myography experiments.

	d_{100}, µm	Maximal force, mN	Relaxation, %
Series of experiment (shown on Figure 4A)			
Solvent (n=6)	286±6	7.4±0.8	3±2
H_2O_2 (n=8)	278±8	6.4±0.6	0±2
Series of experiment (shown on Figure 4B)			
Solvent (n=9)	302±8	11.4±1.5	0±2
SOD+CAT (n=9)	291±9	12.8±1.0	-3±2
Series of experiment (shown on Figure 5A)			
Solvent (n=6)	571±24	31±5	-8±6
DETC (n=6)	617±25	29±5	-7±4
Series of experiment (shown on Figure 5B)			
Solvent (n=6)	248±10	5.4±0.9	9±7
DETC (n=6)	253±11	7.2±1.3	2±1
Series of experiment (shown on Figure 6A)			
Solvent (n=7)	628±13	42±3	78±8
DETC (n=7)	632±21	45±4	87±10
Series of experiment (shown on Figure 6B)			
Solvent (n=7)	250±9	10.8±0.8	100±4
DETC (n=7)	253±10	11.9±1.2	97±3
Series of experiment (shown on Figure 7A)			
Solvent (n=9)	252±7	7.9±0.7	96±8
DETC (n=8)	250±11	8.7±0.7	100±10
L-NNA (n=7)	254±9	8.5±0.7	100±6
L-NNA+DETC (n=8)	260±5	7.9±0.6	99±7
Series of experiment (shown on Figure 7B)			
Solvent (n=7)	236±9	8.1±1.7	85±8
DETC (n=7)	242±15	8.1±1.0	83±8
ODQ (n=8)	237±9	9.3±0.8	95±8
ODQ+DETC (n=8)	241±12	9.0±1.4	98±8
Series of experiment (shown on Figure 7C)			
Solvent (n=7)	237±7	6.3±0.8	3±2
DETC (n=7)	228±8	7.6±1.4	3±2
SNP (n=7)	234±7	6.0±1.2	8±5
SNP+DETC (n=6)	237±8	7.4±0.6	7±5

Negative values reflect the contraction in response to acetylcholine (observed in vessels with removed endothelium).
